# Supplementary material for: Vitamin D Modified DSS-Induced Colitis in Mice via STING Signaling Pathway
Source: Biology (Basel). 2025 Jun 18;14(6):715. doi: 10.3390/biology14060715 (PMC12190092; doi:10.3390/biology14060715)
Supplement: Supplementary file 1 [file biology-14-00715-s001.zip › Supplementary Materials S1: 16s-report of gut microbiota/Groups/diff_analysis/metagenomeRMETACYC/figure/boxplot/top10log.pdf]

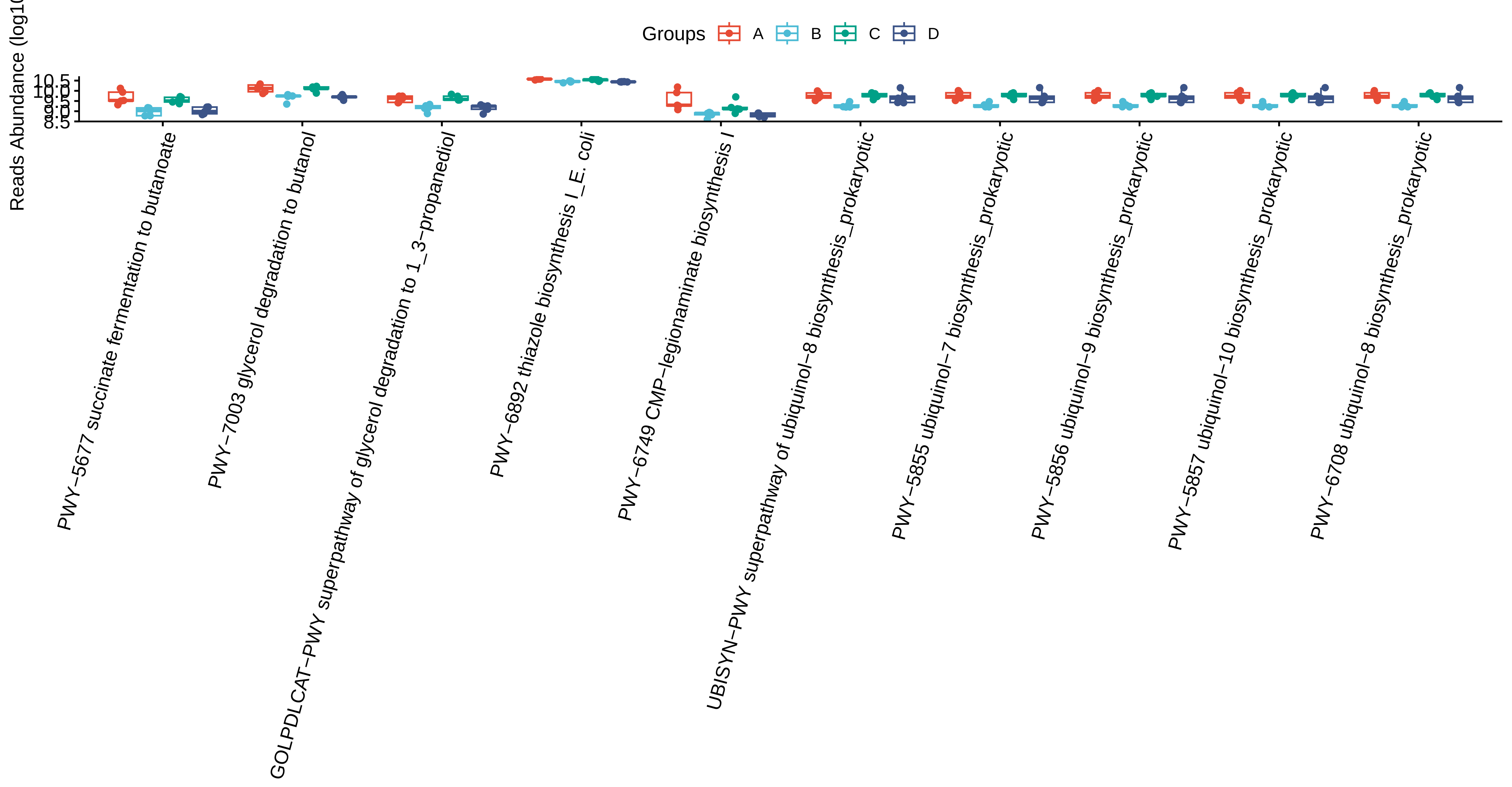

Groups A B C D
Reads Abundance (log10
10.5
10.0
9.5
9.0
8.5
PWY−5677 succinate fermentation to butanoate
PWY−7003 glycerol degradation to butanol
GOLPDLCAT−PWY superpathway of glycerol degradation to 1_3−propanediol
PWY−6892 thiazole biosynthesis I_E. coli
PWY−6749 CMP−legionaminate biosynthesis I
UBISYN−PWY superpathway of ubiquinol−8 biosynthesis_prokaryotic
PWY−5855 ubiquinol−7 biosynthesis_prokaryotic
PWY−5856 ubiquinol−9 biosynthesis_prokaryotic
PWY−5857 ubiquinol−10 biosynthesis_prokaryotic
PWY−6708 ubiquinol−8 biosynthesis_prokaryotic
